# A CellML simulation compiler and code generator using ODE solving schemes

**DOI:** 10.1186/1751-0473-7-11

**Published:** 2012-10-19

**Authors:** Florencio Rusty Punzalan, Yoshiharu Yamashita, Naoki Soejima, Masanari Kawabata, Takao Shimayoshi, Hiroaki Kuwabara, Yoshitoshi Kunieda, Akira Amano

**Affiliations:** 1 Graduate School of Life Sciences, Ritsumeikan University, Shiga, Japan; 2 Graduate School of Information Science and Engineering, Ritsumeikan University, Shiga, Japan; 3 ASTEM Research Institute of Kyoto, Kyoto, Japan

## Abstract

Models written in description languages such as CellML are becoming a popular solution to the handling of complex cellular physiological models in biological function simulations. However, in order to fully simulate a model, boundary conditions and ordinary differential equation (ODE) solving schemes have to be combined with it. Though boundary conditions can be described in CellML, it is difficult to explicitly specify ODE solving schemes using existing tools. In this study, we define an ODE solving scheme description language-based on XML and propose a code generation system for biological function simulations. In the proposed system, biological simulation programs using various ODE solving schemes can be easily generated. We designed a two-stage approach where the system generates the equation set associating the physiological model variable values at a certain time *t* with values at *t* + *Δt* in the first stage. The second stage generates the simulation code for the model. This approach enables the flexible construction of code generation modules that can support complex sets of formulas. We evaluate the relationship between models and their calculation accuracies by simulating complex biological models using various ODE solving schemes. Using the FHN model simulation, results showed good qualitative and quantitative correspondence with the theoretical predictions. Results for the Luo-Rudy 1991 model showed that only first order precision was achieved. In addition, running the generated code in parallel on a GPU made it possible to speed up the calculation time by a factor of 50. The CellML Compiler source code is available for download at http://sourceforge.net/projects/cellmlcompiler.

## Introduction

In recent years, the continued development in computer processing power paved the way for the increased use of biological function simulation. Computers have proven to be invaluable in analysing complex and nonintuitive biological models and biologists are turning to them to complement their experiments. Simulations enable the testing of experimentally unfeasible scenarios and can potentially reduce experimental costs. However, the number and complexity of physiological models has also grown with the increase in computing performance. This creates challenges in reproducing simulated behaviours of the published models and reuse of models by other researchers, hindering the dissemination of science and knowledge integration.

One way to address model complexity is to use markup language-based model descriptions. Some popular examples include CellML [1], SBML (Systems Biology Markup Language) [2] and insilicoML [3]. CellML is an open standard markup language capable of describing mathematical models of cellular functions. SBML is an open interchange machine-readable format for representing models of functions such as metabolism and cell signalling. Meanwhile, insilicoML describes mathematical models for biophysical objects and incorporates morphological information such as shape, angle and position. SED-ML (Simulation Experiment Description Language) [4] is another type of description language which can encode the information of simulation experiments. These markup languages allow researchers to take advantage of the vast amount of biological function models using a common set of easily readable and versatile description rules.

Biological and physiological function models are generally described by differential equations. A typical simulation of biological function models consists of three parts: a model equation, a boundary condition, and an ordinary differential equation (ODE) solver. Model equations and boundary conditions can be described using CellML, while ODE numerical solutions like Euler and Runge-Kutta methods are typically built into the simulation software. However, it is necessary to be flexible in using ODE schemes in order to strike a balance between computational stability and speed. In addition, those using special hardware environments such as massively parallel computer systems require dedicated proprietary software to support their numerical solution needs. Thus, description languages like CellML and dedicated simulation software are not suitable or practical for flexibly incorporating different ODE solving schemes.

To address the need for more flexibility in creating simulation software, we created Time Evolution Calculation Markup Language (TecML), a machine-readable format for encoding ODE numerical solutions. TecML is a description language based on the extensible markup language (XML). This description language is designed to specify and store the numerical methods that can be used for solving the ODEs in biological models. It also allows the assignment of boundary conditions into the simulation experiments. The following sections describe TecML and how it is integrated into the proposed code generation system, which automatically generates codes for biological simulations.

The target of this study is limited to the use of different ODE numerical solutions and their application to models described in CellML. We propose an algorithm that allows users to change the ODE solution and boundary conditions of the model according to the computational needs of their simulation. To verify the effectiveness of the proposed system, we generate executable codes for several CellML models using a number of ODE numerical solutions. The system can generate code in several programming languages and code that runs in both sequential and parallel computing environments. Simulations on GPU (Graphics Processing Units) were undertaken to show the effect of using parallel computing on processing time.

## Biological simulation code generation system

### Summary of simulation code generation system

**Figure 1:**
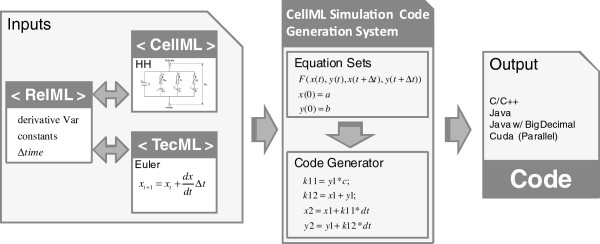
**Code generation system inputs and outputs.** Input and output of the Biological Simulation Code Generation System. The inputs are composed of a CellML, TecML, and RelML file. The system can generate simulation codes in C/C++, Java, and Cuda C programming language.

The proposed method is composed of two stages (Figure 1). In the first stage, the system represents the biological model by incorporating an ODE numerical solution method into the model’s differential equations. This creates the equation sets that calculate the time evolution of the mathematical model. The second stage generates the simulation code for these sets of equations, allowing the user to run computer simulations of the model in machines with general-purpose compilers. This approach enables the flexible construction of code generation modules that can support complex sets of equations. By generating the code separately for each section of the mathematical model, parallel code execution can be easily integrated into the simulation.

To illustrate the capabilities of the proposed system, we used the FitzHugh-Nagumo (FHN) excitable media model [5] as an example. The model, proposed by R. FitzHugh in 1961 and which J. Nagumo et al. created an equivalent circuit, is a simplification of the Hodgkin-Huxley model and can be described by the following equations:

(1) $$\begin{array}{ll} \;r=x^{3}, & \; \end{array}$$

(2) $$\begin{array}{ll} \text{dx}/\text{dt}=x-r/3.0-y+a, & \; \end{array}$$

(3) $$\begin{array}{ll} \text{dy}/\text{dt}=b(x+c-d\cdot y), & \; \end{array}$$

where *r*, *x* and *y* are variables, *t* indicates time and *a, b, c, d* are constants. Removable algebraic expressions such as equation (1) are often used in biological models to improve the model’s readability.

As for the ODE numerical solution, we used the Modified Euler method to solve the ODEs in (2) and (3). By combining a mathematical description of the model above with the ODE solution method and boundary conditions, we can derive the formula to calculate the time evolution of the independent variables *x* and *y*. This involves finding the solution to the variable vector ***ξ*** using its derivatives, temporal variable vector ***ι***, and initial conditions at time *t* given by

(4) $$\begin{array}{ll} d\xi /\text{dt}=f(t,\xi ,\iota ), & \; \end{array}$$

(5) $$\begin{array}{ll} \;\iota =g(t,\xi ). & \; \end{array}$$

The rest of this section shows how the proposed system incorporates the Modified Euler method into the model equations to calculate for ***ξ***. The following set of equations represent the numerical solution with boundary conditions:

(6) $$\begin{array}{ll} \;\xi_{0}=\xi_{t}, & \; \end{array}$$

(7) $$\begin{array}{ll} \;t_{0}=t, & \; \end{array}$$

(8) $$\begin{array}{ll} \;\iota_{0}=g(t_{0},\xi_{0}), & \; \end{array}$$

(9) $$\begin{array}{ll} \;\kappa_{1}=f(t_{0},\xi_{0},\iota_{0}), & \; \end{array}$$

(10) $$\begin{array}{ll} \;\xi_{1}=\xi_{0}+\kappa_{1}\cdot \delta , & \; \end{array}$$

(11) $$\begin{array}{ll} \;t_{1}=t_{0}+\delta , & \; \end{array}$$

(12) $$\begin{array}{ll} \;\iota_{1}=g(t_{1},\xi_{1}), & \; \end{array}$$

(13) $$\begin{array}{ll} \;\kappa_{2}=f(t_{1},\xi_{1},\iota_{1}), & \; \end{array}$$

(14) $$\begin{array}{ll} \;\xi_{2}=\xi_{0}+\frac{1}{2}(\kappa_{1}+\kappa_{2})\cdot \delta , & \; \end{array}$$

(15) $$\begin{array}{ll} \xi_{t+\delta }=\xi_{2}, & \; \end{array}$$

where ***ξ***,***κ*** and ***ι*** are the vectors representing the variables and differential equations in the biological model.

By applying the ODE solver described above, we can arrive at the set of equations detailing the numerical solution for the initial value problem of the FHN model. First, from equations (6) and (7), the current values of the differential variables at *t* are assigned as the initial values with

(16) $$\begin{array}{ll} x_{0}=x_{t}, & \; \end{array}$$

(17) $$\begin{array}{ll} y_{0}=y_{t}, & \; \end{array}$$

(18) $$\begin{array}{ll} t_{0}=\mathrm{t.} & \; \end{array}$$

Then with equations (8) and (9), the differential and nondifferential equations are expanded and evaluated using these initial values to obtain

(19) $$\begin{array}{ll} \;r_{0}=g_{1}(t_{0},{\left[x_{0}\;y_{0}\right]}^{T})=x_{0}^{3}, & \; \end{array}$$

(20) $$\begin{array}{lll} \kappa_{1,x} & =f_{1}(t_{0},{\left[x_{0}\;y_{0}\right]}^{T},r_{0})\; & \;\\ & =x_{0}-r_{0}/3.0-y_{0}+a,\; \end{array}$$

(21) $$\begin{array}{lll} \kappa_{1,y} & =f_{2}(t_{0},{\left[x_{0}\;y_{0}\right]}^{T},r_{0})\; & \;\\ & =b(x_{0}+c-d\cdot y_{0}).\; \end{array}$$

Next, the new values of *x* and *y* are computed as functions of ***κ***_1 _and *δ* (equations (10) and (11)) and given by

(22) $$\begin{array}{ll} x_{1}=x_{0}+\kappa_{1,x}\cdot \delta , & \; \end{array}$$

(23) $$\begin{array}{ll} y_{1}=y_{0}+\kappa_{1,y}\cdot \delta , & \; \end{array}$$

(24) $$\begin{array}{ll} t_{1}=t_{0}+\mathrm{\delta .} & \; \end{array}$$

This process is repeated for *r*_1 _and ***κ***_2 _as shown in equations (12) and (13);

(25) $$\begin{array}{ll} \;r_{1}=g_{1}(t_{1},{\left[x_{1}\;y_{1}\right]}^{T})=x_{1}^{3}, & \; \end{array}$$

(26) $$\begin{array}{lll} \kappa_{2,x} & =f_{1}(t_{1},{\left[x_{1}\;y_{1}\right]}^{T},r_{1})\; & \;\\ & =x_{1}-r_{1}/3.0-y_{1}+a,\; \end{array}$$

(27) $$\begin{array}{lll} \kappa_{2,y} & =f_{2}(t_{1},{\left[x_{1}\;y_{1}\right]}^{t},r_{1})\; & \;\\ & =b(x_{1}+c-d\cdot y_{1}).\; \end{array}$$

Finally, the value of the differential variables are obtained by advancing the solution from time *t* to *t* + *δ* (equations (14) and (15)) with

(28) $$\begin{array}{ll} x_{2}=x_{0}+\frac{1}{2}(\kappa_{1,x}+\kappa_{2,x})\cdot \delta , & \; \end{array}$$

(29) $$\begin{array}{ll} y_{2}=y_{0}+\frac{1}{2}(\kappa_{1,y}+\kappa_{2,y})\cdot \delta , & \; \end{array}$$

(30) $$\begin{array}{ll} x_{t+\delta }=x_{2}, & \; \end{array}$$

(31) $$\begin{array}{ll} y_{t+\delta }=y_{2}. & \; \end{array}$$

The variable *r* of the equation set corresponds to ***ι*** while *x* and *y* are the variables in the vector ***ξ***. The derivatives *dx*/*dt* and *dy*/*dt* are expressed by ***κ*** in equations (20), (21), (26) and (27).

In order to automatically incorporate the ODE numerical solution into the CellML model, we introduced an ODE solving scheme description language called Time Evolution Calculation Markup Language (TecML). TecML is an XML-based description language that can be used to configure ODE solutions and implement the algorithm described in equations (6) – (15). Another input of the system is the Relation Markup Language (RelML) file. The basic role of a RelML file is to relate the variables and variable types of the CellML file into their equivalent in the TecML file.

The simulation code generation system uses these inputs to generate the set of equations describing the time evolution of the variables in biological models. This step creates equations (16) – (31) in the FHN example. Once the model equations are applied with the ODE numerical solution method, the next stage involves the generation of the executable code that will do the actual calculations.

### Description language

The code generation system requires three inputs, namely, the CellML file, TecML file and RelML file. This section gives a short introduction of each and how it is used in the algorithm and consequently, in the FHN model example.

#### CellML model encoding standard

**Figure 2:**
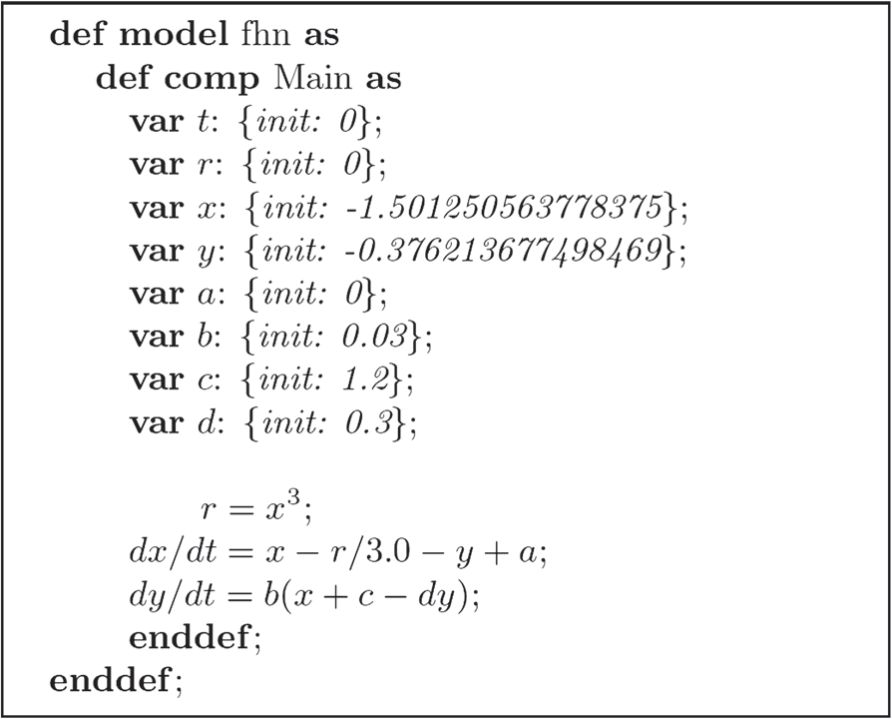
Information in the CellML file of the FHN model (represented in Alan Garny’s COR notation).

The CellML [1] language is an open standard based on XML for describing mathematical models. It was designed to allow scientists to share models even if they are using different model-building software. The majority of the models stored in its repository are cell representations and these include information about the cell structure, equations for underlying processes and in some cases, boundary conditions. Figure 2 shows how a CellML model describes the mathematical equations of the FHN cell model. The file lists the input and output variables as well as their initial values and underlying equations. Note that compact syntax representation is used to show its contents and is based on Alan Garny’s notation for CellML in the Cellular Open Resource (COR) software [6].

#### TecML: an ODE solving scheme description language

**Figure 3:**
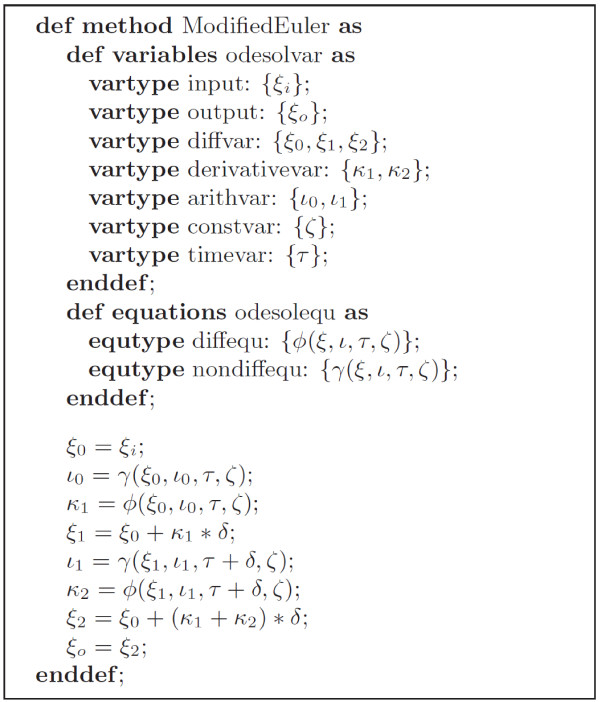
Information in the TecML file of the Modified Euler method where *ξ* is the *diffvar, κ* is the *derivativevar, ζ* is the *constvar,* and variable type *ι* is the *arithvar.*

**Figure 4:**
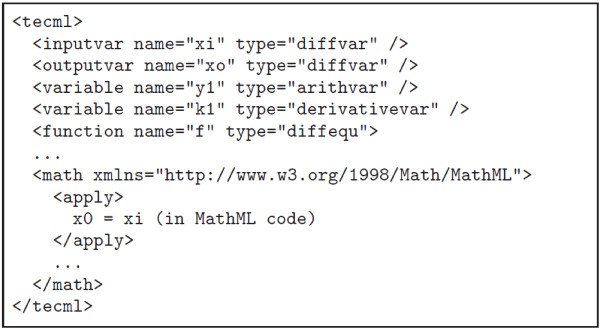
TecML file example.

TecML (Time Evolution Calculation Markup Language) is an XML-based description language designed to describe ODE numerical solutions that can be used in biosimulations. This proposed standard allows the mathematical description of solving schemes like the Euler and Runge-Kutta methods. It allows integration of numerical methods with other description languages like CellML. TecML categorizes variables into six different types (Table 1) namely, *diffvar*, *derivativevar*, *arithvar*, *constvar*, *timevar*, and *deltatimevar*. The variables determined by a rate of change with respect to time (***ξ***) are referred to as differential variables (*diffvar*) while their derivatives (***κ***) are called derivative variables (*derivativevar*). The removable variables (***ι***) are the arithmetic variables (*arithvar*) and variables that do not change in value (***ζ***) are the constants (*constvar*). In addition, the time (*t*) and time increment (*δ*) are referred to as *timevar* and *deltatimevar*, respectively. TecML also divides the mathematical equations into two types; namely, differential (***f***()) and non-differential (***g***()) equations. Equations of type (*diffequ*) are the derivatives of a function while (*nondiffequ*) are the arithmetic functions. The information and example of a TecML file for the Modified Euler method are shown in Figure 3 and Figure 4, respectively.

**Table 1:** Variable and function types in TecML

**Type**	**Definition**
*diffvar*	Differential variable (variable is a function of time)
*arithvar*	Temporal variable (can be substituted with math equations)
*derivativevar*	Derivative of diffvar
*constvar*	Constants
*timevar*	Time variable
*deltatimevar*	Variable denoting the change of time per step
*diffequ*	Differential equation
*nondiffequ*	Non-differential equation

#### Relation Markup Language (RelML)

**Figure 5:**
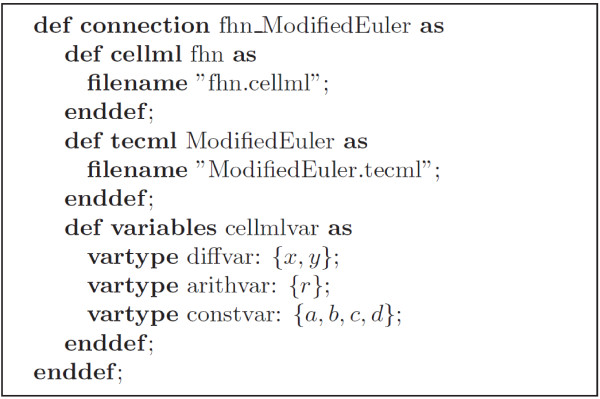
Information contained in the RelML file for the FHN model and Modified Euler method.

RelML is a language for describing the correspondence between the variables in the CellML model file and the variable types in an ODE numerical solution scheme described in a TecML file. Figure 5 shows the correspondence of variables described in the FHN CellML file (Figure 2) and the variable types in the TecML file (Figure 3). For the FHN model in Figure 2, variables *x* and *y* are defined as *diffvar* and their respective derivatives *dx*/*dt* and *dy*/*dt* as *derivativevar*. In addition, *a, b, c,* and *d* are *constvar* type while *r* is considered a temporal variable or *arithvar* type. Equations (2) and (3) are the formulas for calculating the *diffvar* so the functions are categorized as *diffequ* while the arithmetic equation for *r* in equation (1) is a *nondiffequ*.

**Figure 6:**
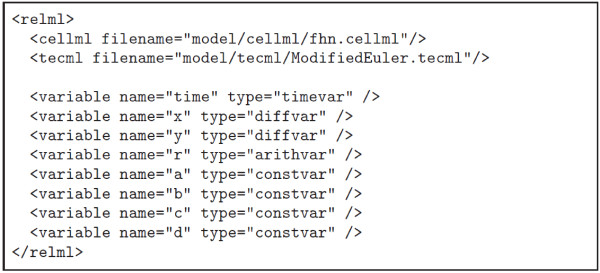
RelML file example for FHN model and Modified Euler method.

An example RelML file where the Modified Euler method is applied to the FHN model is shown in Figure 6. The first two statements after the header indicate the filename and location of the corresponding CellML and TecML file. The succeeding lines enumerate all the variables used in the FHN model and their corresponding types. The complete RelML and TecML files used in this paper have been published on the Web [7].

### Executable code generation for equation sets

The system implementation of the code generator allows the creation of codes in a number of programming languages. Table 2 lists the tested inputs and outputs of the system. For a desktop CPU environment, the generator can produce source codes in C, Java, and Java with BigDecimal library, which can handle higher precision computing. Codes can be generated for both single cell and cell array simulation. The cell array code generator can produce both 1D and 2D excitation propagation codes. The generated codes for 2D simulation describes the excitation propagation in a rectangular array with *N*×*M* number of cells. Aside from generating single CPU codes, it can also create codes suited for parallel computing that runs on a GPU machine.

**Table 2:** Input and output code types in the system

	
Cell Physiology	FitzHugh-Nagumo model [5]
Model	LuoRudy 1991 model [8], Kyoto 2003 Model [9]
ODE Solution	Euler Method, Modified Euler Method
Scheme	1^*st*^-, 2^*nd*^-, 4^*th*^-order Runge-Kutta Method
Generated codes	C code, Java, Java with BigDecimal
	Cuda code (GPU)

## Methods: simulation code generation algorithm

### Cell model description

The cell model is a collection of variables and equation and can be written as

(32) $$\left\{\begin{array}{c} k=dx/\text{dt}=f(x,y,t,z)\\ y=g(x,y,t,z) \end{array}\;,\right)$$

where ***x*** is a differential variables vector, ***k*** is a derivatives vector, ***y*** is a temporal variables vector, and ***z*** is a constants vector while *t* is the scalar variable for time. These variable vectors can be expressed as sets with

(33) $$\begin{array}{ll} x=\;{\left[x_{1},x_{2},\cdots \;,x_{N_{x}}\right]}^{T}, & \; \end{array}$$

(34) $$\begin{array}{ll} k=\;{\left[k_{1},k_{2},\cdots \;,k_{N_{x}}\right]}^{T}, & \; \end{array}$$

(35) $$\begin{array}{ll} y=\;{\left[y_{1},y_{2},\cdots \;,y_{N_{y}}\right]}^{T}, & \; \end{array}$$

(36) $$\begin{array}{ll} z=\;{\left[z_{1},z_{2},\cdots \;,z_{N_{z}}\right]}^{T}, & \; \end{array}$$

where *N*_*x*_,*N*_*y*_,*N*_*z*_are the variable count for ***k***,***y***, and ***z***, respectively. Furthermore, ***f***(***x***,***y***,*t*,***z***) and ***g***(***x***,***y***,*t*,***z***) are function vectors with *k*_*i*_ = *f*_*i*_(***x***,***y***,*t*,***z***) and *y*_*i*_ = *g*_*i*_(***x***,***y***,*t*,***z***). These function vectors are defined by

(37) $$\begin{array}{cccc} f(x,y,t,z) & = & {\left[f_{1}(x,y,t,z),\cdots \;,f_{N_{x}}(x,y,t,z)\right]}^{T}, & \end{array}$$

(38) $$\begin{array}{cccc} g(x,y,t,z) & = & {\left[g_{1}(x,y,t,z),\cdots \;,g_{N_{y}}(x,y,t,z)\right]}^{T}. & \end{array}$$

### ODE solving scheme

Table 3 shows the elements inside a TecML file. Note that all the TecML variables are denoted with Greek letters. The differential equations for the cell model are represented by the terms *d****ξ***/*dt* = ***Φ***(***ξ***,***ι***,*t*,***ζ***) and ***ι*** = ***Γ***(***ξ***,***ι***,*t*,***ζ***). Inside a TecML file, the dependence between the differential variables ***ξ***_**0**_(time *t*) and $\left(\xi_{N_{\xi }}\right)$ (time *t* + *δ*) is given by

(39) $$\begin{array}{ll} \xi_{i}=\sigma_{i}(\Xi ,\pi ,\delta )\;(0\leq i\leq N_{\xi }), & \; \end{array}$$

(40) $$\begin{array}{ll} \kappa_{i+1}=\Phi (\xi_{i},\iota_{i},\tau_{i},\zeta )\;(0\leq i\leq N_{\xi }), & \; \end{array}$$

(41) $$\begin{array}{ll} \iota_{i}=\Gamma (\xi_{i},\iota_{i},\tau_{i},\zeta )\;(0\leq i\leq N_{\xi }), & \; \end{array}$$

(42) $$\begin{array}{ll} \tau_{i}=T_{i}(t,\delta )\;(0\leq i\leq N_{\xi }), & \; \end{array}$$

**Table 3:** Information written in a TecML file

**Notation**	**Definition**
$\left(\xi_{0}={\left[\xi_{0,1},\xi_{0,2},\cdots \;,\xi_{0,N_{x}}\right]}^{T}\right)$	Input differential variable vector
$\left(\xi_{N_{\xi }}={\left[\xi_{N_{\xi },1},\xi_{N_{\xi },2},\cdots \;,\xi_{N_{\xi },N_{x}}\right]}^{T}\right)$	Output differential variable vector
***ξ***_*i*_(1 ≤ *i* ≤ *N*_*ξ*_)	Time differential variable vector
$\left(\Xi ={\left[\xi_{0},\xi_{1},\cdots \;,\xi_{N_{\xi }}\right]}^{T}\right)$	Differential variable vector of ***ξ***
*t*	Current time value
*δ*	Time step
$\left(\zeta ={\left[\zeta_{0},\zeta_{1},\cdots \;,\zeta_{N_{z}}\right]}^{T}\right)$	Constants vector
*τ* = *T*_*i*_(*t*,*δ*)	Calculation of variable *T*_*i*_
$\left(\begin{array}{c} \Phi (\xi ,\iota ,\tau ,\zeta )=[\phi_{1}(\xi ,\iota ,\tau ,\zeta ),\\ {\cdots \;,\phi_{N_{x}}(\xi ,\iota ,t,\zeta )]}^{T} \end{array}\right)$	Differential equation vector
$\left(\begin{array}{c} \Gamma (\gamma ,\iota ,\tau ,\zeta )=[\gamma_{1}(\xi ,\iota ,\tau ,\zeta ),\\ {\cdots \;,\gamma_{N_{y}}(\xi ,\iota ,t,\zeta )]}^{T} \end{array}\right)$	Temporal function vector
***κ***_*i* + 1_ = ***Φ***(***ξ***,***ι***,*t*,***ζ***)	Derivative variable vector
***ι***_*i*_ = ***Γ***(***ξ***,***ι***,*t*,***ζ***)	Temporal variable derived from ***ξ***
$\left(\pi ={\left[\kappa_{1},\kappa_{2},\cdots \;,\kappa_{N_{\xi }}\right]}^{T}\right)$	Derivative vector of ***κ***
*ξ*_*i*_ = *σ*(Ξ,K,δ)	Relation between *ξ*_*i*_and Ξ,K,δ

where the differential variable vector ***Ξ*** and derivative variable vector ***π*** are given by

(43) $$\begin{array}{ll} \Xi ={\left[\xi_{0},\xi_{1},\cdots \;,\xi_{N_{\xi }}\right]}^{T}, & \; \end{array}$$

(44) $$\begin{array}{ll} \pi ={\left[\kappa_{1},\kappa_{2},\cdots \;,\kappa_{N_{\xi }}\right]}^{T}. & \; \end{array}$$

### CellML and TecML integration

The integration of the CellML model and TecML solving scheme involves the mapping of corresponding variables. The mapping shows how each physiological model variable written in CellML is replaced with its corresponding TecML variable. The differential variable vector ***ξ*** of TecML corresponds to the differential variable vector ***x*** of CellML and reads as

(45) $$\xi_{k}\leftarrow x_{k}={\left[x_{k,1},x_{k,2}\cdots x_{k,N_{x}}\right]}^{T}.$$

TecML’s derivative variable ***κ*** equates to the CellML’s derivative variable *d****x***/*dt* and the temporal variable ***ι*** corresponds to ***y*** and given by

(46) $$\begin{array}{ll} \kappa_{k+1}\leftarrow dx_{k}/\mathrm{dt}={\left[\frac{dx_{k,1}}{\mathrm{dt}},\frac{dx_{k,2}}{\mathrm{dt}},\cdots \;,\frac{dx_{k,N_{x}}}{\mathrm{dt}}\right]}^{T}, & \; \end{array}$$

(47) $$\begin{array}{ll} \iota_{k}\leftarrow y_{k}={\left[y_{k,1},y_{k,2},\cdots \;,y_{k,N_{y}}\right]}^{T}. & \; \end{array}$$

Furthermore, the TecML equations ***κ*** = ***Φ***(***ξ***,***ι***,*t*,***ζ***) and ***ι*** = ***Γ***(***ξ***,***ι***,*t*,***ζ***) correspond to the CellML equations ***f*** and ***g***, respectively, as shown by

(48) $$\begin{array}{ll} \Phi (\xi ,\iota ,\tau ,\zeta ) & \leftarrow f(x,y,t,z),\; \end{array}$$

(49) $$\begin{array}{ll} \Gamma (\xi ,\iota ,\tau ,\zeta ) & \leftarrow g(x,y,t,z).\; \end{array}$$

### Replacement algorithm

Once the variables are mapped, the differential and arithmetic equations are transformed and expanded according to the numerical method described in the TecML file. First, the variables in the CellML model equations are replaced with their corresponding TecML variables. Each model equation is searched for any *diffvar*, *derivativevar*, *arithvar*, *constvar*, *timevar*, or *deltatimevar* and that variable is replaced with the corresponding TecML variable name. This replacement procedure is represented in Strachey brackets and expressed by the following function:

(50) $$\begin{array}{ll} R_{v}⟦\text{equ}⟧ & =R_{x}⟦R_{k}⟦R_{y}⟦R_{t}⟦R_{z}⟦R_{d}⟦\text{equ}⟧⟧⟧⟧⟧⟧,\; \end{array}$$

where ⟦*equ*⟧ is the TecML equation and, for all *i* = 1,2,…,*N*_*ξ*_, the replacement functions are

(51) $$\begin{array}{ll} R_{x}⟦\xi_{i}⟧=⟦x_{i}⟧, & \; \end{array}$$

(52) $$\begin{array}{ll} R_{k}⟦\kappa_{i}⟧=⟦k_{i}⟧, & \; \end{array}$$

(53) $$\begin{array}{ll} R_{y}⟦\iota_{i}⟧=⟦y_{i}⟧, & \; \end{array}$$

(54) $$\begin{array}{ll} R_{t}⟦\tau_{i}⟧=⟦t_{i}⟧, & \; \end{array}$$

(55) $$\begin{array}{ll} R_{z}⟦\zeta ⟧=⟦z⟧, & \; \end{array}$$

(56) $$\begin{array}{ll} R_{d}\mathrm{⟦\delta ⟧}=\mathrm{⟦d⟧.} & \; \end{array}$$

Note that the Strachey brackets in $\begin{array}{l} R_{x}⟦\xi_{i}⟧=⟦x_{i}⟧ \end{array}$means that all the TecML *diffvar* (*i.e.* for *i* = 1…*N*_*ξ*_) in the argument is replaced with the corresponding CellML *diffvar****x***_*i*_. This function is true for all the other variable types in TecML.

**Figure 7:**
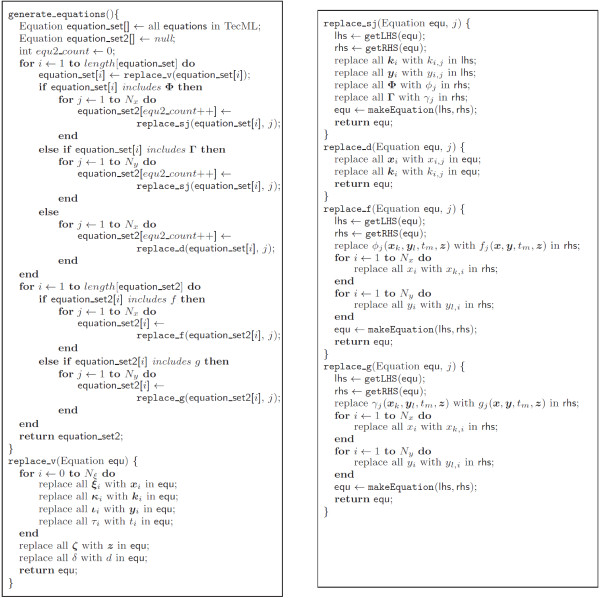
Algorithm for generating the set of ODE numerical solution equations.

The generated set of equations advances the solution of the differential variables from time *t* to *t* + *δ*. Figure 7 shows how the algorithm generates the simulation program from the input files. Note that the subroutine replace_v() in the algorithm replaces variables in the CellML model equation with the TecML variables as expressed in equation (50). The function replace_sj() generates one scalar equation from a vector equation and appends index j to the variables. In the equations which do not contain functions *f * or *g*, replace_d() generates multiple scalar equations from a vector equation. Finally, replace_f() and replace_g() unfold functions *f * and *g*, respectively. These subroutines can be expressed in the following transformations:

(57) $$\begin{array}{c} \;R_{s,j}\mathrm{⟦equ⟧}=\text{makeEquation}⟦S_{y,j}⟦S_{k,j}⟦\text{getLHS}\mathrm{⟦equ⟧⟧⟧},\\ \;\;\;S_{g,j}⟦S_{f,j}⟦\text{getRHS}\mathrm{⟦equ⟧⟧⟧⟧}, \end{array}$$

(58) $$\begin{array}{ll} R_{d,j}⟦\text{equ}⟧=S_{k,j}⟦S_{x,j}⟦\text{equ}⟧⟧, & \; \end{array}$$

(59) $$\begin{array}{ll} R_{f}⟦\phi_{j}(\xi ,\iota ,\tau ,\zeta )⟧=T_{y,l}⟦T_{x,k}⟦f_{j}(x,y,t,z)\mathrm{⟧⟧}, & \; \end{array}$$

(60) $$\begin{array}{ll} R_{g}⟦\gamma_{j}(\xi ,\iota ,\tau ,\zeta )⟧=T_{y,l}⟦T_{x,k}⟦g_{j}(x,y,t,z)\mathrm{⟧⟧}, & \; \end{array}$$

where the index-appending functions $\left(S,\;\right)$and unfolding functions $\left(T,\;\right)$are defined as

(61) $$\begin{array}{ll} S_{x,j}⟦x_{i}⟧=⟦x_{i,j}⟧, & \; \end{array}$$

(62) $$\begin{array}{ll} S_{y,j}⟦y_{i}⟧=⟦y_{i,j}⟧, & \; \end{array}$$

(63) $$\begin{array}{ll} S_{f,j}⟦\Phi ⟧=⟦\phi_{j}⟧, & \; \end{array}$$

(64) $$\begin{array}{ll} S_{g,j}⟦\Gamma ⟧=⟦\gamma_{j}⟧, & \; \end{array}$$

(65) $$\begin{array}{ll} T_{x,k}⟦x⟧=⟦x_{k}⟧, & \; \end{array}$$

(66) $$\begin{array}{ll} T_{y,k}⟦y⟧=⟦y_{k}\mathrm{⟧.} & \; \end{array}$$

## Experiments and results

### Code generation for the cell model simulation

The FHN cell model [5] was used to evaluate the proposed system. The system was also used to generate simulation codes for a number of cell models (LuoRudy1991 [8], LuoRudy1994 [10] and KyotoModel2003 [9]) and with other more accurate ODE numerical methods (e.g. 4th-order Runge-Kutta method). All the generated code used in the simulation experiments can be downloaded from the files section of the program generator site [7].

The traditional approach to creating simulation experiment codes also offers little flexibility once the software is created. Changing the numerical ODE solution method means making major revisions in the simulation code. The proposed method allows the flexibility of using different simulation models, boundary conditions, and numerical methods for a simulation experiment. Since the simulation codes are generated automatically, users can choose or change their desired ODE solver without making changes in the simulation codes themselves. Also, this can give clear information on what is calculated to generate the simulation results.

The different cell models and ODE numerical methods produced varying simulation code sizes. Table 4 lists the number of execution steps generated for the cell models using different ODE solutions. The table shows that the more complex the model becomes and the more equations it has, the larger the number of steps to compute the model equations in the code.

**Table 4:** The number of execution steps for the generated codes for different cell models and ODE numerical methods

**Cell Model**	**Euler**	**Modified Euler**	**Runge-Kutta**
FHN	11	16	26
LuoRudy1991	70	117	211
LuoRudy1994	123	211	387
Kyoto Model	335	560	1200

An issue in approximating ODE solutions is the accuracy of the numerical method used. A good approximation to the underlying differential equation needs to be achieved in order to arrive at accurate simulation results. We tested a number of commonly used ODE numerical methods to determine how the use of different solutions affect the accuracy of the calculations. The three methods used were Euler, Modified Euler and 4th-order Runge-Kutta. Each of these methods were used to generate an FHN model simulation code that runs in a single CPU. The generated code can run in different compilers and does not require third party software.

**Figure 8:**
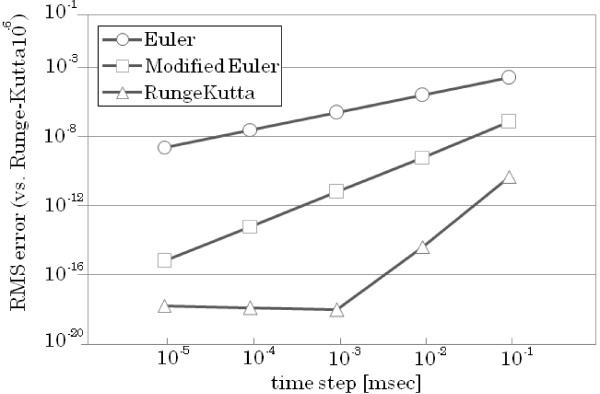
**Relationship between the time step and calculation error for each ODE numerical method used in generating the FHN model.** The results and root-mean-square errors (RMSE) are computed using Java BigDecimal. The result of the Runge-Kutta method with 10^-6 ^ms time step is used as reference for the RMSE computations.

In order to test the accuracy of the ODE numerical methods, simulation codes were generated in Java using the BigDecimal class and numeric formatting. The Java BigDecimal can represent a large number of decimal places and help avoid rounding errors. It can offer higher precisions than the 16 decimal digits offered by floating point *double*. In the simulation codes, we used BigDecimal to represent the numbers in a 32-digit decimal point precision format for all the ODE solving methods. Different time steps were also used in testing the accuracy of these ODE methods, ranging from 10^−1 ^ms to 10^−5 ^ms. The simulation using a time step of 10^−6 ^and Runge-Kutta as ODE solving scheme was used as the basis to compute for the root-mean-square error (RMSE) and evaluate the accuracy of the other calculations. The RMSE was computed for all the calculations using different ODE numerical methods and in varying time steps (Figure 8). Each level in the RMSE indicates a 1/10 less accuracy in the simulation results compared to the calculations using Runge-Kutta and 10^−6 ^ms time step.

The first-order Euler method gave the largest error (log error ≈10^−3^) while the Modified Euler resulted to a smaller error. The fourth-order Runge-Kutta method resulted with the best accuracy (log error ≈10^−10^). However, the Runge-Kutta error is almost constant from the time step 10^−3 ^ms, . This can be attributed to the rounding error of digits over the used 32-digit precision.

**Figure 9:**
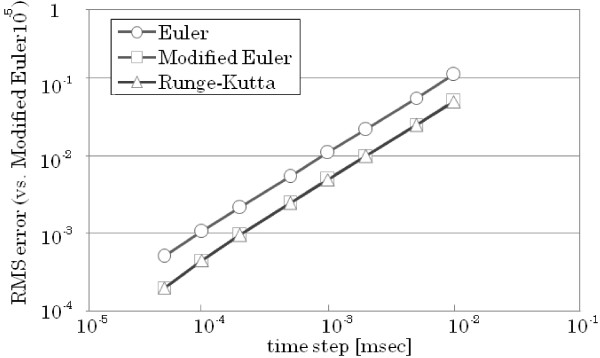
Relationship between the time step and calculation error for each ODE numerical method used in generating the LuoRudy 1991 model.

Simulation codes using different ODE numerical methods were also generated for models more complicated than the FHN or Hodgkin-Huxley model. For this, we used the model introduced by C. Luo and Y. Rudy in 1991 [8]. It is a simple cell model of cardiac action potential that uses Hodgkin-Huxley type equations to calculate ionic currents. The RMSE computations undertaken for the FHN model were also applied for the Luo-Rudy 1991 simulations. Codes were generated for the three ODE solutions with time steps ranging from 10^−1 ^to 10^−4 ^ms. Meanwhile, the simulation using the Euler method with a time step of 10^−5 ^ms was used as the reference for RMSE calculations (Figure 9).

### Excitation propagation simulations

One possible application of 1- or 2-dimensional excitation propagation simulation is cardiac arrhythmia research. Computational models have provided new insights into the underlying mechanisms of re-entry like the role of ionic currents and ion channel mutations [11]. In addition, designs of defibrillation treatments can be optimized using simulations of excitation propagation to achieve good clinical results for patients.

Simulation codes for the Luo-Rudy 1991 model were generated to simulate cell excitation propagation on a 2D homogeneous sheet. The two-dimensional cell action potential propagation simulations were run for both CPU and GPU to compare the speed of calculations. The CPU simulation was performed with an intel Core i7 880 processor with 8 GB of memory running Windows 7. The GPU has a single Nvidia Tesla C2050 processor with 448 CUDA cores, and 3 GB memory in a CentOS 5.5 system. The programs are written in C for the CPU simulations and Cuda C for the GPU.

In the experiments, the size of the homogeneous sheet was varied from 10240 (10240 × 1) to 1433600 (10240 × 14) cells with 10240-cell increments. The computation time was measured for each increment. Figure 9 shows the CPU and GPU computational time at different cell array sizes. Results showed that by using the GPU, the computational time can be accelerated 50 times as compared to using a standard CPU.

## Discussion

Traditionally, biological function simulation programs have to be manually created from scratch. This is manageable with small models or simulation experiments but it becomes less practical once model complexity increases. The more complex the model becomes the larger the program becomes and the harder it is to create and maintain such programs. The Doty model [12] showed that the cost of software development is proportional to the square exponential of the number of computational steps. As seen from Table 4, the number of computational steps increases with the complexity of the model. By using this system, the creation of a simulation program from biological models becomes simpler. The system takes input from experts in biological functions through CellML models and mathematical experts through ODE numerical solutions and automatically generates the simulation code. The automatic code generation allows it to deal with large and complex models without the proportional increase in software development cost. This also keeps the software maintenance cost low since the programs are created with the same structure, regardless of the biological model under study.

The system’s support for multiple programming languages makes it easier for users to test the model in different programming environments and keep the cost of switching from one programming language to a low. Based on the codes generated for the purpose of this paper, science and engineering students typically create a Java simulation code from the C version within four days.

The system requires the formatted RelML file to do the first stage of the algorithm and the RelML information can also be used to automatically determine the boundary conditions. The RelML information can be extracted automatically from the CellML model file but boundary conditions are not always available. We need a mathematical representation of boundary conditions which are compatible with declarative description. This can be addressed by the inclusion of PEPML experimental protocol [13] in the system. PEPML is an XML-based language that can describe the initial conditions and procedures of an experimental protocol. It describes boundary conditions in a mathematical form and is purely represented in a declarative manner, making it easy to combine with the current implementation of our system.

**Figure 10:**
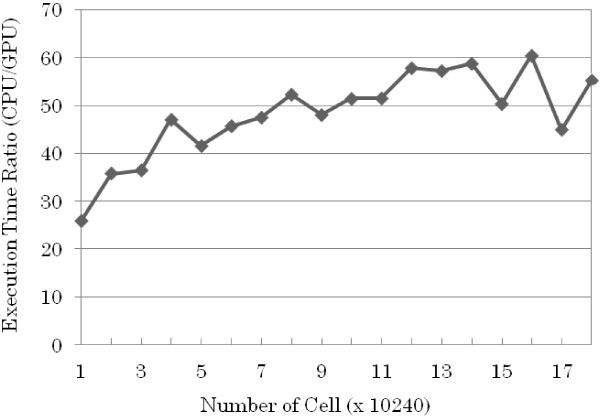
CPU and GPU execution time ratio of the excitation propagation simulation.

The attainable order of accuracy for approximating the Luo-Rudy 1991 model equations was determined by running simulations of the model using different ODE numerical methods. The results of the comparisons between ODE solving schemes showed that the model can be accurately predicted by the first-order ODE solutions. The use of higher-order numerical solutions has a minimal effect on the values of the approximations (Figure 10). The Luo-Rudy model is a set of nonlinear differential equations that use higher order ODE solutions but it can be seen from the Taylor series expansion that equations from the second-order term has little or negligible effect. The structure of the system, where the ODE solver can be easily changed with the appropriate TecML file, allows one to confirm the accuracy of the model simulation such as the case with the Luo-Rudy 1991 model (see Additional file 1).

Although the system is fully implemented, further improvements are still to be added. The generated excitation propagation code does not consider the optimization of the model variables for parallel processing. Therefore, code parallelization is low and may have contributed to slower execution in the GPU environment. Also, the system can only handle equations with a single term in the left-hand side. Other equation forms will be addressed in future implementations.

Furthermore, simultaneous calculations using differential algebraic equations (DAE) are still not available. Simultaneous equations appear in some models and implicit numerical methods. The handling of implicit methods is available in the inputs but not in the current implementation of our system. A simultaneous equation library, other ODE numerical solutions library like CVODE, or a linear algebra library like LINPACK could be incorporated into our system. This can be a possibility with our plan to design a library interface description language to handle numerical solution inputs.

Future implementations also need to address the need for a declarative design of describing procedural methods. Complex ODE numerical methods like the ones described in the Kinetic Simulation Algorithm Ontology (KiSAO) [14] are difficult or impossible to encode in the current version of TecML. We are in the process of redesigning TecML and the system to incorporate methods that are suitable for the types of problems scientists are addressing with manually-created code, including adaptive methods. This can greatly enhance the usability of the proposed method.

In addition, the system may be integrated with SED-ML. SED-ML allows description of the simulation environment, which includes the name of the numerical method to be used in the simulation. A starting point for integration would be to provide TecML information of a numerical method for the KiSAO entry in SED-ML to allow simulation software to refer to this description. Another point would be to create support for SED-ML files in the code generation system in order to create more complex experiments that use more than one model or experiments with different simulation methods applied. This will allow users the flexibility of choosing not only the ODE solving methods but also the experiment protocol when generating their simulation codes.

## Conclusion

In this paper, we proposed a method to automatically generate executable simulation codes using CellML physiological models and ODE numerical solution methods. The generated code describes the time-evolution of the set of differential equations enumerated by the CellML model. The code generation system is composed of a two-stage approach that allows flexible generation of complex sets of equations.

To evaluate the effectiveness of the proposed system, several combinations of physiological cell models and ODE solving schemes were generated. The output of the numerical approximations were in accordance with the published results of the cell models. Results for the Luo-Rudy 1991 simulations also indicated that it only has first-order accuracy. The comparison of execution time for 2D excitation propagation also showed that the use of GPU can accelerate the processing time by 50 times as compared to a CPU.

The code generation system allows executable simulation codes to be easily generated from CellML and TecML files. This can be very useful in the field of biological model simulation since it provides the tools to quantitatively evaluate the mathematical equations in these models.

## Competing interests

The authors declare that they have no competing interests.

## Author’s contributions

FRP has been involved in the drafting of the manuscript, programming and gathering of experimental data. YY was responsible for programming of the code generator software and gathering data. NS was the main programmer of the code generator. MK was involved in the simulation experiments and data gathering as well as programming. TS, HK and YK acted as consultants for the algorithm and system design. AA has been the main architect of the algorithms and system design and has given the final approval of the version to be published. All authors read and approved the final manuscript.

## Supplementary Material

Additional file 1 Appendix. Click here for file
