# Supplementary material for: A CellML simulation compiler and code generator using ODE solving schemes
Source: Source Code Biol Med. 2012 Oct 19;7:11. doi: 10.1186/1751-0473-7-11 (PMC3778851; doi:10.1186/1751-0473-7-11)
Supplement: Additional file 1 — Appendix. [file 1751-0473-7-11-S1.pdf]

# A APPENDIX

## A.1 TecML and RelML Files

The following shows the full version of one of the TecML (Fig. 1) and RelML (Fig. 2) files used in the FHN simulation experiments.

```
<tecml>
 <inputvar name="xi" type="diffvar" />
   <outputvar name="xo" type="diffvar" />
   <variable name="d" type="deltatimevar" />
   <variable name="t" type="timevar" />
   <variable name="k1" type="derivativevar" />
   <variable name="k2" type="derivativevar" />
   <variable name="x0" type="diffvar" />
   <variable name="x1" type="diffvar" />
   <variable name="x2" type="diffvar" />
   <variable name="y0" type="arithvar" />
   <variable name="y1" type="arithvar" />
   <variable name="z" type="constvar" />
   <function name="g" type="nondiffequ">
        <argument type="diffvar" />
        <argument type="timevar" />
        <argument type="arithvar" />
        <argument type="constvar" />
   </function>
   <function name="f" type="diffequ">
        <argument type="diffvar" />
        <argument type="timevar" />
        <argument type="arithvar" />
        <argument type="constvar" />
   </function>

   <math xmlns="http://www.w3.org/1998/Math/MathML">
        <apply><eq/>
           <ci>x0</ci>
           <ci>xi</ci>
        </apply>
        <apply><eq/>
           <ci>y0</ci>
           <apply>
                <fn><ci>g</ci></fn>
                <ci>x0</ci>
                <ci>t</ci>
                <ci>y0</ci>
                <ci>z</ci>
           </apply>
        </apply>
        <apply><eq/>
           <ci>k1</ci>
           <apply>
                <fn><ci>f</ci></fn>
                <ci>x0</ci>
                <ci>t</ci>
                <ci>y0</ci>
                <ci>z</ci>
           </apply>
        </apply>
        <apply><eq/>
           <ci>x1</ci>
           <apply><plus/>
                <ci>x0</ci>
                <apply><times/>
                   <ci>k1</ci>
                   <ci>d</ci>
                </apply>
           </apply>
        </apply>
```

Fig. 1: TecML file for the Modified Euler method.

```
        <apply><eq/>
           <ci>y1</ci>
           <apply>
                <fn><ci>g</ci></fn>
                <ci>x1</ci>
                <apply><plus/>
                   <ci>t</ci>
                   <ci>d</ci>
                </apply>
                <ci>y1</ci>
                <ci>z</ci>
           </apply>
        </apply>
        <apply><eq/>
           <ci>k2</ci>
           <apply>
                <fn><ci>f</ci></fn>
                <ci>x1</ci>
                <apply><plus/>
                   <ci>t</ci>
                   <ci>d</ci>
                </apply>
                <ci>y1</ci>
                <ci>z</ci>
           </apply>
        </apply>
        <apply><eq/>
           <ci>x2</ci>
           <apply><plus/>
                <ci>x0</ci>
                <apply><times/>
                   <apply><divide/>
                        <ci>d</ci>
                        <cn>2</cn>
                   </apply>
                   <apply><plus/>
                        <ci>k1</ci>
                        <ci>k2</ci>
                   </apply>
                </apply>
           </apply>
        </apply>
        <apply><eq/>
           <ci>xo</ci>
           <ci>x2</ci>
        </apply>
   </math>

</tecml>
```

Fig. 1: TecML file cont...

```
<relml>
   <cellml filename="model/cellml/FHN.cellml"/>
   <tecml filename="model/tecml/ModifiedEuler.tecml"/>

   <variable name="time" type="timevar" />
   <variable name="x" type="diffvar" />
   <variable name="y" type="diffvar" />
   <variable name="r" type="arithvar" />
   <variable name="a" type="constvar" />
   <variable name="b" type="constvar" />
   <variable name="c" type="constvar" />
   <variable name="d" type="constvar" />
</relml>
```

Fig. 2: RelML file for the FHN simulation with Modified Euler method as the ODE solving scheme.

## A.2 Luo-Rudy 1991 Cell Model Analysis

In order to validate the accuracy of the simulated code for the Luo-Rudy 1991 model, we compared the numerical results of the code to a mathematical analysis of the model. The second-order Taylor series analysis was used for the analytical results.

The Luo-Rudy model expressed the rate of change of cell membrane potential ($V_m$) as:

$$\frac{dV_m}{dt} = -\frac{1}{C_m}(I_{stim} + I_{ion}) \tag{1}$$

where $C_m$ is the membrane capacitance, $I_{st}$ is the stimulus current and $I_{ion}$ is the total membrane ionic current. The model contains 6 ionic currents that are determined by 6 gating variables. Each ionic current $I_y$ is a function of $V_m$ and some combination of gating variables ($\boldsymbol{P} = \{m, h, j, d, f, X, V_m\}$). Letting $C_m = 1$, $dV_m/dt$ can be expressed as:

$$\begin{aligned}\frac{dV_m}{dt} &= -[I_{stim} + I_{Na}(\boldsymbol{P}) + I_{si}(\boldsymbol{P}) + I_K(\boldsymbol{P}) \\ &\quad + I_{K1}(\boldsymbol{P}) + I_{Kp}(\boldsymbol{P}) + I_b(\boldsymbol{P})] \end{aligned} \tag{2}$$

where $I_{Na}, I_{si}, I_K, I_{K1}, I_{Kp}$ and $I_b$ are the ionic currents.

To see if the model is approximately first-order, we derived its Taylor series expansion and compute for its second- to first-order term ratio. The equation for the second-order Taylor series expansion of a real function $f(t)$ about a point $t = a$ is given by

$$f(t) = f(a) + f'(a)(t-a) + \frac{f''(a)}{2!}(t-a)^2 \tag{3}$$

Letting $f(t) = V_m(t)$ and $a = 0$, the second-order Taylor expansion of the membrane potential $V_m$ with respect to time $t$ can be expressed by

$$V_m(t) = V_m(0) + \frac{dV_m}{dt}(t) + \frac{1}{2}\frac{d^2V_m}{dt^2}(t)^2 \tag{4}$$

To get the ratio between the first and second order term in the series, we must solve for the second derivative of $V_m$ with respect to $t$ ($d^2V_m/dt^2$).

$$\begin{aligned}\frac{d^2V_m}{dt^2} &= -\frac{d}{dt}[I_{stim} + I_{Na}(\boldsymbol{P}) + I_{si}(\boldsymbol{P}) + I_K(\boldsymbol{P}) \\ &\quad + I_{K1}(\boldsymbol{P}) + I_{Kp}(\boldsymbol{P}) + I_b(\boldsymbol{P})] \end{aligned} \tag{5}$$

Here, we assigned $I_{stim} = 0$. The derivative of the other terms in the right hand side of the equation depends on multiple gating variables. The derivative of ionic current $I_y$, assuming that all gate variables in $\boldsymbol{P}$ vary with time $t$, can be derived using the total derivative in the form of

$$\frac{df_{I_y}}{dt} = \frac{\partial f_{I_y}}{\partial t} + \sum_{i=1}^{n}\frac{\partial f_{I_y}}{\partial P_i}\cdot\frac{dP_i}{dt} \tag{6}$$

where $f_{I_y}$ is the ionic current function and $P_i$ is the gating variable. Using this form to solve for the derivative of one of the ionic currents, $I_{Na}$ with $p_{Na} = \{m, h, j, V\}$, we get

**Table 1.** Derivative of the ionic currents in the Luo-Rudy 1991 model.

| $I_{ion}$ Type | Derivative |
|---|---|
| $I_{Na}$ | $69m^2hj(V_m - 54.79)\frac{dm}{dt} + 23m^3j(V_m - 54.79)\frac{dh}{dt} + 23m^3h(V_m - 54.79)\frac{dj}{dt} + 23m^3hj\frac{dV_m}{dt}$ |
| $I_{si}$ | $0.09f(V_m - 117.51)\frac{dd}{dt} + 0.09d(V_m - 117.51)\frac{df}{dt} + 0.09df\frac{dV_m}{dt}$ |
| $I_K$ | $0.196(V_m + 77.57)\frac{dX}{dt} + 0.196X\frac{dV_m}{dt}$ |
| $I_{K1}$ | $0.292\frac{dV_m}{dt}$ |
| $I_{Kp}$ | $4.25 \times 10^{-9}\frac{dV_m}{dt}$ |
| $I_b$ | $0.0392\frac{dV_m}{dt}$ |

$$\frac{dI_{Na}}{dt} = \sum_{i=1}^{7}\frac{\partial\left(23m^3hj(V_m - E_{Na})\right)}{\partial P_i}\cdot\frac{dP_i}{dt} \tag{7}$$

$$\begin{aligned} &= 69m^2hj(V_m - E_{Na})\frac{dm}{dt} + 23m^3j(V_m - E_{Na})\frac{dh}{dt} \\ &\quad + 23m^3h(V_m - E_{Na})\frac{dh}{dt} + 23^3mhj\frac{dV_m}{dt} \end{aligned} \tag{8}$$

The rest of the ionic currents in (5) can be solved using the procedure in equations (7) – (8). Table 1 shows the resulting equations for the ionic current derivatives, expressed in terms of the membrane voltage and gating variable derivatives. The total of these derivatives is equal to the second derivative of $V_m$. The graph of the first-

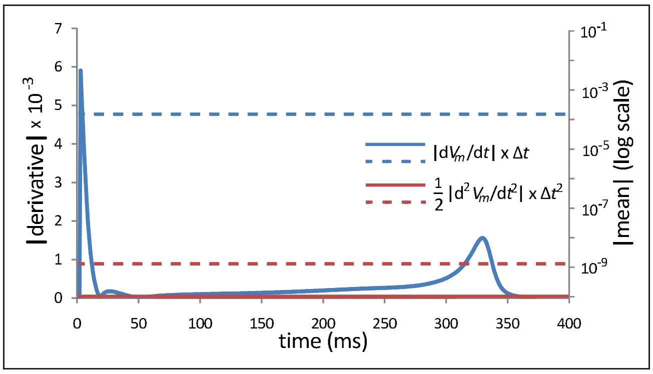


Fig. 3: Simulation results for the first and second term of the Taylor series for the Luo-Rudy 1991 model. The mean value (logarithmic scale) of the terms are shown in dashed lines.

($\frac{dV_m}{dt} \times \Delta t$) and second-order ($\frac{1}{2}\frac{d^2V_m}{dt^2} \times \Delta t^2$) term in the Taylor series expansion are shown in Fig. 3. The second-to-first-order term ratio, which has a mean value of $7.84 \times 10^{-6}$ (0.000784%), confirms that the the value of the second-order term is much smaller than the first. This validates the experimental results showing the Luo-Rudy 1991 model as a first-order model.
